# Chemical Defense by the Native Winter Ant (*Prenolepis imparis*) against the Invasive Argentine Ant (*Linepithema humile*)

**DOI:** 10.1371/journal.pone.0018717

**Published:** 2011-04-19

**Authors:** Trevor R. Sorrells, Leah Y. Kuritzky, Peter G. Kauhanen, Katherine Fitzgerald, Shelby J. Sturgis, Jimmy Chen, Cheri A. Dijamco, Kimberly N. Basurto, Deborah M. Gordon

**Affiliations:** Department of Biology, Stanford University, Stanford, California, United States of America; University of Plymouth, United Kingdom

## Abstract

The invasive Argentine ant (*Linepithema humile*) is established worldwide and displaces native ant species. In northern California, however, the native winter ant (*Prenolepis imparis*) persists in invaded areas. We found that in aggressive interactions between the two species, *P. imparis* employs a potent defensive secretion. Field observations were conducted at *P. imparis* nest sites both in the presence and absence of *L. humile*. These observations suggested and laboratory assays confirmed that *P. imparis* workers are more likely to secrete when outnumbered by *L. humile*. Workers of *P. imparis* were also more likely to secrete near their nest entrances than when foraging on trees. One-on-one laboratory trials showed that the *P. imparis* secretion is highly lethal to *L. humile*, causing 79% mortality. The nonpolar fraction of the secretion was chemically analyzed with gas chromatography/mass spectrometry, and found to be composed of long-chain and cyclic hydrocarbons. Chemical analysis of dissected *P. imparis* workers showed that the nonpolar fraction is derived from the Dufour's gland. Based on these conclusions, we hypothesize that this chemical defense may help *P. imparis* to resist displacement by *L. humile*.

## Introduction

The Argentine ant (*Linepithema humile*) is a common invasive species worldwide in regions with mediterranean or subtropical climates like much of California [1]. Once established, *L. humile* causes the decrease of populations of many organisms in its invaded range, including plants [2], other arthropods [3], [4] and even vertebrates [5]. *L. humile* drastically reduces populations of many native ant species [6], [7] due to both exploitative and interference competition [4], [8], [9].

The native winter ant, *Prenolepis imparis*, has been found to persist in the presence of the invasive Argentine ant in northern California [7], [8], [10]. Both species tend scale insects and consume their excretions [11]–[13]. Populations of *L. humile* rely more heavily on the use of scale insects as they become more established in invaded areas [14]. Workers of *P. imparis* are slightly larger than those of *L. humile,* but retreat from encounters with *L. humile* at bait [15]. As its common name implies, *P. imparis* is more active at low temperatures [11], [16], while *L. humile* activity peaks in the summer. Coexistence of *L. humile* and *P. imparis* has thus been explained by temporal niche separation [6], [7], [17], [18]. However, because *L. humile* and *P. imparis* are both active throughout the year (KF, unpublished data), temporal partitioning cannot be the only explanation for the persistence of *P. imparis* in areas invaded by *L. humile*.

**Figure 1:**
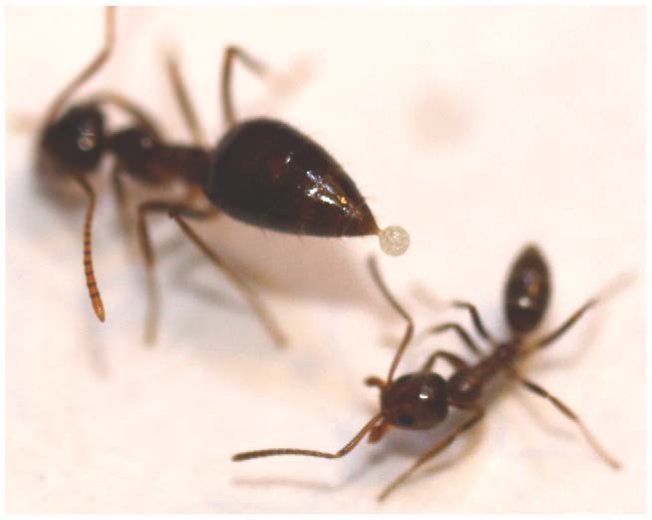
The *P. imparis* secretion. A single *P. imparis* worker is shown with a liquid droplet containing bubbles at the tip of its raised abdomen. The secretion is then applied to the body of the *L. humile* ant.

Use of chemical defense compounds in ants may promote coexistence of species with otherwise mismatched interference and exploitation competitive abilities. The alkaloid repellent chemicals of *Monomorium* species, for example, are postulated to be the mechanism that allows for their coexistence with the bigger, faster moving, and much more aggressive *Iridomyrmex* species in Australia [19]. We observed that in aggressive interactions, *P. imparis* secretes an opaque white liquid from the abdomen, which is often aimed at or deposited on *L. humile* workers (Fig. 1). Lynch, et al. [20] and Fellers [21] observed this *P. imparis* behavior in encounters with other native species, and noted that this substance causes an "immediate loss of coordination in the victim."Other ant species show similar chemical defenses, including the projection of formic acid sprays by *Cataglyphis* species in aggressive interactions [22], the use of dienal poisons secreted from the abdomen of species of Crematogaster [23], and a chemical deterrent that allows *Formicoxenus* parasites to avoid attack from their hosts [24].

In this investigation, we asked: 1) What is the frequency and context of the chemical secretion used by *P. imparis*? 2) How does the use of chemical secretion by *P. imparis* against *L. humile* depend on the relative numbers of each species present? 3) Does the use of the chemical secretion by *P. imparis* depend on proximity of the *P. imparis* ant to its nest? 4) What is the effect of the *P. imparis* secretion on *L. humile* mortality? 5) What is the chemical composition of the *P. imparis* secretion?

## Methods

### Frequency and Context of *P. imparis* Aggression Towards *L. humile*

Observations of *L. humile* and *P. imparis* were conducted in landscaped areas of the Stanford University campus in northern California, U.S.A. (37°25′48″N, 122°10′12″W, altitude 29 m). Eight 0.25-m^2^ quadrats were established at the base of trees surrounded by mulch, leaves, or lawn. Each tree had a *P. imparis* nest within 1–2 m. Observations were made for 5-min intervals within 2 hours after sunrise and sunset between 5–30 Nov 2007. Ant species and ant density(classified as 1–10, 11–50, 51–150, or >150 ants in the quadrat)were recorded. There were a total of 237 5-min observations.

We recorded the incidence of aggressive behavioral interactions that occurred between a *P. imparis* worker and an *L. humile* worker. These behaviors included (1) gaster-flagging: the ant raises its abdomen erect in the air [25], (2) secretion of an opaque liquid from the abdomen, (3) chasing: two ants meet, then one quickly pursues the other and (4) fighting: one ant bites the legs, antennae, or body of another ant. We assessed the relationship between ant density and behavior using a generalized linear mixed model with the lmer function from lme4 package in R. Because behavior was recorded as counts, each independent variable was assumed to be distributed as a Poisson random variable. Density of *P. imparis* and of *L. humile* were included as fixed factors, and site was included as a random factor. Because the two ant species have seasonal fluctuations in activity, we included observation date as a third fixed factor. The full model also included interactions between all variables. Ant density was treated in the model as the lowest number of ants in each density category (1, 11, 51, and 151). We then performed model selection using the Akaike information criterion (AIC) [26].

### Effect of Relative Numbers of *P. imparis* and *L. humile*

In our field observations, we could not formally exclude the possibility that an additional, unknown variable, such as food availability, caused the relationship found between ant density and behavior. To control for such variables, we conducted assays to examine further the effects of the relative numbers of *L. humile* and *P. imparis* on aggression. On 10 days in April and May 2008, ants were collected from five different sites on Stanford University campus, each with nests of the two species within 50 m of one another. Experiments were performed outdoors within 10 minutes of collecting the ants. We combined 20 ants in a 10 cm diameter arena with *P. imparis* proportions of 0.2 (4 *P. imparis* 16 *L. humile*), 0.5 (10 *P. imparis* 10 *L. humile*), and 0.8 (16 *P. imparis* 4 *L. humile*). Each site-proportion combination was assayed three times and on at least two different days for a total of 45 trials. In our preliminary observations, ants of both species became agitated upon collection, and cooling decreased this agitation. Although other studies have found cooling to alter aggression in unpredictable ways [27], we saw no other effects of cooling and no intraspecific aggression in preliminary trials. Separate vials of the two species were cooled on ice to calm the ants before they were tapped into the dish. Ants were observed for 5 minutes and aggressive behavior was recorded as described above.

In experiments in which there were unequal numbers of the two species (*P. imparis* proportions of 0.2 and 0.8), we normalized counts of behavior to the probability that one ant encountered an ant of the other species. Assuming that the encounter rate is random, this probability is proportional to the product of the number of ants of each species present. We normalized the behavior counts in trials with *P. imparis* proportions of 0.2 and 0.8 by multiplying the behavior count by (10×10)/(16×4) = 1.5625. The data were then analyzed with generalized linear mixed models. Each behavior was analyzed with a separate model. The normalized behavior counts were treated as Poisson-distributed dependent variables, the independent variable was proportion of *P. imparis*, and site was included as a random factor. Significance was assessed using a sequential Bonferroni correction for multiple comparisons with an alpha level of 0.05. To calculate the mean number of secretions per *P. imparis* per trial, we divided the average number of secretions in a trial by the number of *P. imparis* ants.

### Effect of Proximity to *P. imparis* Nest on Incidence of Secretion

To compare secretion rates by *P. imparis* workers when near the nest or far from the nest, we measured secretion rate of *P. imparis* by workers within 30 cm of nests at three nests, and on four trees that were at least 2 m from nests. Individual ants were prodded for 2–3 s to determine if the ant could be induced to secrete. Each ant was then aspirated to prevent double counting and the propagation of alarm pheromones. Trials were performed for 20 ants on each of the four trees and near the three nests, for a total of 140 trials on two days in April 2009. We compared the number of trials in which workers secreted near the nest or on trees away from nests using Fisher's exact test.

### Effect of *P. imparis* Secretion on *L. humile* Mortality

To test the effect of the *P. imparis* secretion on *L. humile*, we conducted laboratory assays using a dissecting microscope on 4 days between May and August 2009. One worker ant of each species was transferred to a 1 cm diameter arena and the two ants were observed for up to 180 s. If an aggressive interaction occurred, we observed the ants for another 60 s or until the *P. imparis* secreted a liquid from its abdomen. After a *P. imparis* worker secreted, it was aspirated from the arena, and the behavior of the *L. humile* ant was observed for 4 min. We recorded limbs lost during the initial observation and mortality after 1 hr for both species. We used a chi-square test to determine whether mortality of *L. humile* workers following trials in which *P. imparis* secreted differed from mortality following trials in which *P. imparis* did not secrete.

### Chemical Analysis of *P. imparis* Secretion

*Prenolepis imparis* workers were collected from three different nests on the Stanford University campus. Ants were collected from as close to the nest entrance as possible. One to five ants from a nest were placed into a Petri dish and agitated with the tip of a glass pipette until one of the ants secreted a white substance from the tip of its abdomen. When the secretion landed on the pipette tip, it was immediately dissolved into 50 uL of 100% pentane (Sigma, St. Louis, Missouri, U.S.A.). Secretions that landed on any surface other than the pipette tip were discarded to avoid contamination. Between 10 and 20 secretions were collected for each 50 uL sample. When individual ants secreted multiple times, each secretion was collected, dissolved, and counted as separate in the sample.

Additional ants were collected for dissection to determine the anatomical source of the secretions. Ants collected for dissection were placed at −20°C immediately after capture. *Prenolepis imparis* workers were dissected in distilled water under a Wild Heerbrugg M5A dissecting microscope (Wild Heerbrugg AG, Switzerland). The Dufour's gland, venom sac, and acidopore with the Dufour's gland and venom sac attached were dissected from 5–10 ants, and each was separately extracted in 2 mL of 100% pentane overnight at 4°C. The suspension solution was taken up and run through 1 ml of a silica gel (28–200 mesh, Sigma, St. Louis, Missouri, U.S.A.) column with a 1 mL wash of 100% pentane. The eluent was dried overnight and redissolved in 50 uL of pentane.

All samples were analyzed at the Vincent Coates Foundation Mass Spectrometry Laboratory, Stanford University Mass Spectrometry (http://mass-spec.stanford.edu). Gas chromatography-mass spectrometry (GC/MS) analysis was performed using a 6890/5973 GC/MS (Agilent Technologies, Santa Clara, California, U.S.A.) equipped with electronic pressure control, split/splitless inlet, and 7683 autosampler. Separations were done on a 250 um ID×30 m length, 25 um stationary phase thickness HP-MS5 capillary column (Agilent Technologies), with helium as the carrier gas at 1 mL/min. One uL injections were made at a 5∶1 split ratio with pressure pulse of 30 psi. The GC oven temperature was held at 60°C for 5 min, ramped at 20°C/min to 200°C, and held for 5 minutes. The MS was operated in full scan mode, from 50 to 550 amu. Results were analyzed using the spectral library that was part of the available GC/MS software, and relevant peaks were identified and compared across 14 samples: 5 samples from live secretions, 3 from dissected Dufour's glands, 3 from venom sacs, and 3 from the dissected acidipores with Dufour's gland and venom sac attached. Both polar and nonpolar fractions of the secretions were extracted, but only the nonpolar fraction produced conclusive results using GC/MS, so our analysis focuses on this fraction.

## Results

### Context of *P. imparis* and *L. humile* Aggression in Field Observations

**Figure 2:**
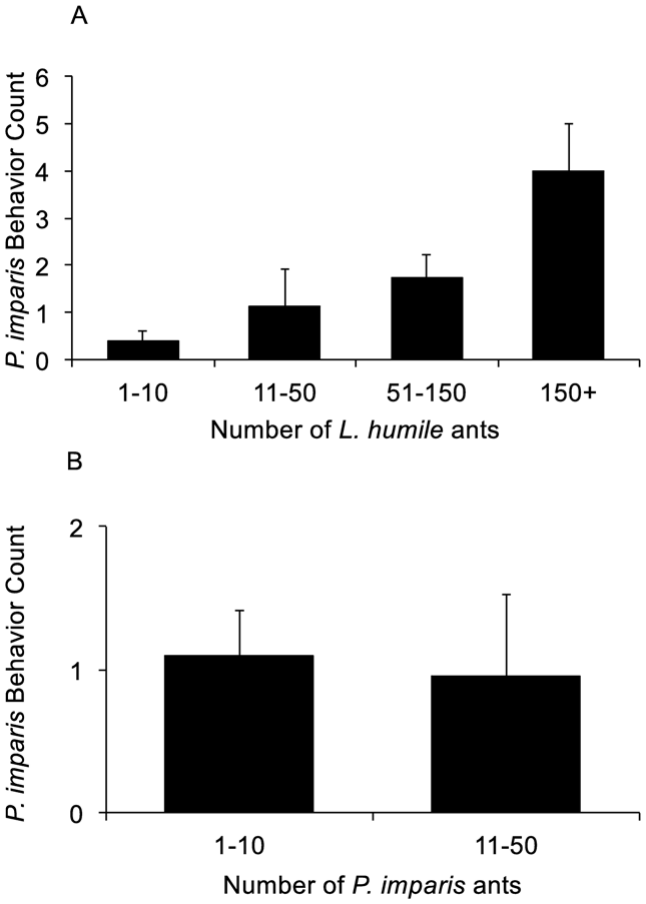
Aggressive interactions in field observations of *P. imparis* and *L. humile*. Shown are the mean numbers of aggressive interactions by A. *L. humile* density and B. *P. imparis* density. Error bars show standard error of the mean. Sample sizes in A were *N* = 22 for 1–10, *N* = 16 for 11–50, *N* = 4 for 51–150, and *N* = 2 for 150+. Sample sizes in B were *N* = 21 for 1–10 and *N* = 23 for 11–50.

The amount of aggression between *P. imparis* and *L. humile* depended on the numbers of workers of each species (Fig. 2), the time of year, all two-way interactions, and the three-way interaction between these variables; no terms were removed from the full model during model selection (Generalized Linear Mixed Model, AIC = 86.3). Aggressive behavior between *P. imparis* and *L. humile* was more likely in the presence of higher numbers of *L. humile* (GLMM, ΔAIC = 29.5) and higher numbers of *P. imparis* (GLMM, ΔAIC = 11.1). The interaction between these variables showed that at low numbers of *L. humile*, aggression was less likely at high numbers of *P. imparis* (GLMM, ΔAIC = 18.0). Instances of aggressive behavior decreased over the month of observations (GLMM, ΔAIC = 17.0). The interactions between the variables *P. imparis* density and time (GLMM, ΔAIC = 2.9), *L. humile* density and time (GLMM, ΔAIC  = 10.1), and the three-way interaction (GLMM, ΔAIC = 15.6) also significantly improved the model. The effects of *P. imparis* density, *L. humile* density, and their interaction were strongest earlier in the season, and weakend over time. The many significant interactions may be due to the small sample size at high *L. humile* densities.

We observed three non-aggressive behaviors that occurred only among *P. imparis* workers: (1) clustering: a large group of ants remains huddled in a single location within the quadrat throughout the observation, (2) twitching: two or more ants stand close together and lurch repeatedly in unison, (3) mandible-clasping: two ants grasp their jaws together for a period of time; this may have been trophallaxis. We recorded a total of 57 instances of aggressive behavior and 308 instances of non-aggressive behavior in 237 observation periods. Secretion by *P. imparis* occurred 12 times during five separate observations, one of which occurred at a site with only *P. imparis*.

### Effect of Relative Numbers of *P. imparis* and *L. humile* in Fighting Assays

**Figure 3:**
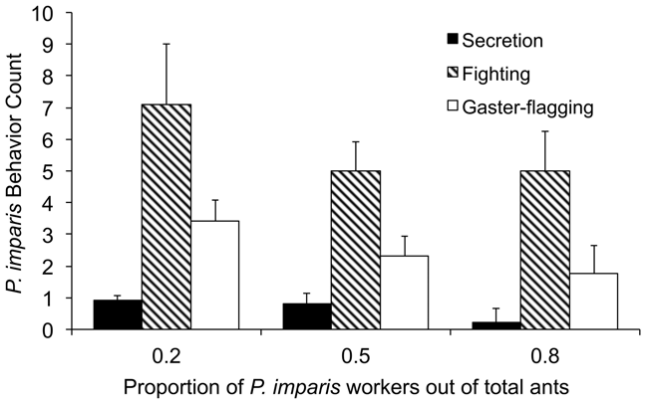
Effect of the relative numbers of *L. humile* and *P. imparis* on aggressive behavior. Shown are the mean number of observations per trial of secretion (filled bars), gaster-flagging (hatched bars), and fighting (open bars) in relation to the proportion of *P. imparis* workers. Each assay was performed with 20 total *P. imparis* and *L. humile* workers and *N* = 15 trials for each proportion. Error bars show standard error of the mean.

Workers of *P. imparis* were more likely to behave aggressively and to deploy their chemical secretion when heavily outnumbered by *L. humile*. In controlled aggression assays, aggressive behavior increased as the proportion of *P. imparis* decreased and the proportion of *L. humile* increased (Fig. 3) for gaster-flagging (GLMM, *Z* = −2.84, *N* = 45, *P* = 0.005), fighting (GLMM, *Z* = −2.33, *N* = 45, *P* = 0.020), and secretion (GLMM, *Z* = −2.39, *N*  = 45, *P*  = 0.017). *P. imparis* secreted on any body part of the *L. humile* worker. The number of secretions per *P. imparis* worker was 0.0083 in trials with a proportion of *P. imparis* of 0.8, 0.08 in trials with a proportion of 0.5, and 0.15 in trials with a proportion of 0.2.

### Effect of Distance from *P. imparis* Nest on Incidence of Secretion

When agitated by prodding with a metal wire, *P. imparis* in the field readily secreted. Workers secreted in 42% of occasions when prodded within 30 cm of the nest and 1% of occasions when prodded while they were foraging on trees 1–2 m away from the nest (Fisher's exact test, *P*<<0.001).

### Effect of *P. imparis* Secretion on *L. humile* Mortality

**Figure 4:**
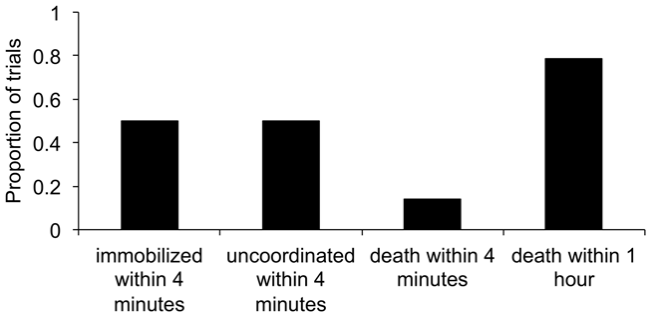
Effect of chemical secretion on *L. humile*. Proportion of *L. humile* workers that demonstrated specific behaviors after contact with the *P. imparis* secretion. There were *N* = 14 trials in which the *P. imparis* ant secreted on the *L. humile* ant.

Secretion by *P. imparis* often killed *L. humile* ants (Fig. 4). Secretions by *P. imparis* were opaque, filled with small bubbles, and usually secreted directly onto the body of the *L. humile* worker. In one-on-one trials, 79% of *L. humile* ants died within an hour when contacted by the *P. imparis* secretion (*X* ^2^_1_  = 14.97, *P* <0.001). When there was aggressive behavior but no contact with the secretion, no *L. humile* workers died. In the 29 trials, the *P. imparis* ant secreted once in six trials, twice in five trials, and three times in three trials. The *L. humile* ant died in four out of six trials in which the *P. imparis* ant secreted only once, and in two trials the *L. humile* ant died almost immediately after being contacted by the secretion. In 12 out of 14 trials, the *L. humile* ant was immobilized or walked without coordination immediately following contact with the secretion. The *P. imparis* lost one or more limbs in 11 of the 22 trials in which fighting occurred. Loss of limbs by *P. imparis* was associated with secretion by *P. imparis* (*X* ^2^_1_ = 4.91, *P* = 0.027).

### Chemical Analysis of *P. imparis* Secretion

We analyzed the nonpolar fractions of the *P. imparis* secretion. The analysis yielded a mixture of long, straight-chain and cyclo- alkanes and alkenes, and were derived anatomically from the Dufour's gland [28], [29]. The most abundant of these compounds were hexadecene (10.51–15.76% Area), octadecene (5.36–8.47% Area), tetradecene (5.61–7.20% Area), tetradecane (4.75–36%Area), octylcyclohexane (4.11–4.66% Area), decylcyclohexane (3.59–5.61% Area ), hexadecane (3.24–5.01% Area), and dodecylcyclohexane (1.96–3.64% Area). The compounds identified were present in both live secretions and in dissected Dufourvs glands.

## Discussion

The chemical defensive behavior of *P. imparis* is an effective weapon against *L. humile*. We found that the secretion used by *P. imparis* usually resulted in injury and death of *L. humile* workers. The more *P. imparis* was outnumbered by *L. humile*, the more likely it was to deploy its secretion. Individual *P. imparis* ants often secreted many times in our assays. One secretion was sufficient to kill or severely impair a single *L. humile* ant. Thus, a single *P. imparis* ant may be capable of killing many *L. humile* ants.

We found that, as in other species [30], *P. imparis* workers are more likely to deploy their chemical defense in encounters near their nest than in encounters while foraging on trees. This indicates that *P. imparis* modifies its behavior according to the value of the resource it is defending. This may be because the production of the secretion is metabolically costly.

The use of a lethal secretion may help *P. imparis* defend against *L. humile*, which has been shown to raid the nests of native ants [4]. We occasionally observed *L. humile* trails that led to *P. imparis* nests, which caused *P. imparis* to position workers just inside the nest entrance with their abdomens pointed outward. Native ant colonies under attack frequently plug and aggressively defend their nest entrances [4], [31], [32].

The outcomes of interspecific conflict are strongly influenced by colony size [33]; large numbers of small ants can prevail against smaller numbers of larger ants [34]. Aggressive response may depend on the number of conspecifics present, as well as the number of competitiors [35], [36]. Large population size may, in part, explain the success of *L. humile* as an invader [37]. Both in field observations and in fighting assays, we found that *P. imparis* was more likely to deploy its chemical secretion and other aggressive behavior when heavily outnumbered by Argentine ants (Fig. 2, Fig. 3). The increased aggression by *P. imp*aris may have been in response to the behavior of *L. humile* ants, which are more aggressive when among larger numbers of their own species than in one-on-one encounters [15], [38].

We do not know how often *P. imparis* uses its secretion against conspecifics and other native species. We saw a *P. imparis* worker using its secretion against another *P. imparis* worker once, while use of the secretion against *L. humile* was much more common. Previous studies found *P. imparis* to be behaviorally dominant to native species [20], [21] and it is possible that the secretion contributes to this heirarchy.

The chemical composition of the non-polar portions of the secretion is similar to that of other ant chemical defenses, which are also comprised largely of hydrocarbons of lengths varying from 1 to 20 carbons [28], [39]. Hydrocarbon compounds with greater than 20 carbons may be detected using a GC/MS column that reaches higher temperatures, but these compounds are waxy and are likely to be cuticular hydrocarbons rather than components of the volatile secretion. All but the three cyclic compounds found in *P. imparis* were also present in the secretions of three other formicine ants: *Formica nigricans*, *F. rufa*, and *F. polyctena* [26].

Our analysis confirms the composition of the nonpolar portion of the secretion. We were unable to verify the contents of the polar fraction. Because *P. imparis* is in the formicine subfamily, it is likely that the polar fraction of the secretion is primarily formic acid, as it is in other formicine species [25], [38], [40]. GC/MS could not confirm this, however, and future research is needed to investigate alternative analytical methods that may offer better detection of carboxylic acid species like formic acid that may be present in the polar fraction. Formic acid is commonly used as a chemical defense in ants, and also in other insects, such as some lepidopteran larvae [38], [40]. It has been suggested that the non-polar, long, straight-chain portions of formicine ant secretions may serve as cuticular penetrating agents for formic acid [26], [28]. This could be the function of the *P. imparis* nonpolar fraction, but further analysis is needed to identify which portion of the secretion causes mortality.

Our results show that *P. imparis* can attack and kill *L. humile* workers with a potent chemical weapon. The Argentine ant is successful at displacing most native species in invaded areas [6]–[8], but several native species are likely to persist, by avoiding competition or through agonistic behavior [7], [9], [41], [42]. Previous observations suggested that the ability of *P. imparis* to persist in areas invaded by *L. humile*, despite competition for the honeydew of scale insects, was due to seasonal temporal partitioning [6], [7], [17] which can facilitate coexistence [43]. Although the two species differ in seasonal peaks of activity, *P. imparis* and *L. humile* are active throughout the year, and we observed frequent interactions between the species when foraging on trees. Active resistance by *P. imparis*, as well as temporal partitioning, may account for its ability to persist in areas invaded by *L. humile*.
